# Movements, Home Range and Site Fidelity of Snapper (*Chrysophrys auratus*) within a Temperate Marine Protected Area

**DOI:** 10.1371/journal.pone.0142454

**Published:** 2015-11-06

**Authors:** David Harasti, Kate A. Lee, Christopher Gallen, Julian M. Hughes, John Stewart

**Affiliations:** 1 Fisheries Research, NSW Department of Primary Industries, Nelson Bay, NSW, 2315, Australia; 2 Department of Biological Sciences, Macquarie University, North Ryde, Sydney, Australia; 3 New South Wales Department of Primary Industries, Sydney Institute of Marine Science, Mosman, New South Wales, 2088, Australia; Universidade Federal do Rio de Janeiro, BRAZIL

## Abstract

Understanding the movement dynamics of marine fish provides valuable information that can assist with species management, particularly regarding protection within marine protected areas (MPAs). We performed an acoustic tagging study implemented within the Port Stephens-Great Lakes Marine Park on the mid-north coast of New South Wales, Australia, to assess the movement patterns, home range and diel activity of snapper (*Chrysophrys auratus*; Sparidae); a species of significant recreational and commercial fishing importance in Australia. The study focused on *C*. *auratus* movements around Cabbage Tree Island, which is predominantly a no-take sanctuary zone (no fishing), with an array of acoustic stations deployed around the island and adjacent reefs and islands. Thirty *C*. *auratus* were tagged with internal acoustic tags in November 2010 with their movements recorded until September 2014. Both adult and juvenile *C*. *auratus* were observed to display strong site fidelity to Cabbage Tree Island with a mean 12-month residency index of 0.83 (range = 0 low to 1 high). Only three fish were detected on acoustic receivers away from Cabbage Tree Island, with one fish moving a considerable distance of ~ 290 kms over a short time frame (46 days). The longest period of residency recorded at the island was for three fish occurring regularly at the site for a period of 1249 days. *Chrysophrys auratus* displayed strong diurnal behaviour and detection frequency was significantly higher during the day than at night; however, there was no significant difference in detection frequency between different hours. This study demonstrates that even small-scale protected areas can benefit *C*. *auratus* during multiple life-history stages as it maintains a small home range and displays strong site fidelity over a period of 3 years.

## Introduction

During the past century, tagging of marine fish has provided information on short-term movements and species home ranges [1], and given an insight into large scale migrations of various species [2–4]. Monitoring of fish movements has been done using various tagging methods, such as conventional ‘dart and anchor’ and elastomer tags [5–7] and satellite tags [8, 9]. More recently, the use of acoustic tags has become a core method for assessing fish movements [10]. During the past decade, advancements in acoustic technologies have led to increased battery life and tag miniaturisation allowing for species to be monitored for much longer periods of time, providing longer term data sets on movements [11]. Movement data collected from acoustic tagging studies can be used to determine site fidelity and species home ranges [12–14] and habitat utilisation [15, 16]. This information may contribute to species protection through the implementation of fishing closures or designs of protected areas [17, 18].

Marine Protected Areas (MPAs) have been implemented worldwide and play important roles in management of fisheries resources, conserving marine biodiversity, protection of sensitive habitats and enhancing eco-tourism [19–21]. Numerous studies have shown MPA’s to be successful in promoting habitat recovery, particularly coral reefs [22, 23], ensuring genetic connectivity [24] and providing economic benefits to local communities through increased tourism and improved fisheries [25–27]. The benefits of MPAs for fish species are well documented [26, 28], with studies indicating numerous fish species to be more abundant and larger in size within no-take protected areas than outside [29–32]. MPAs have been successful in conserving stocks of exploited species and provide benefits to fisheries through the ‘spill-over effect’ [27, 33] and larval replacement across protected area boundaries [34–36]. However, in order for MPAs to be effective in protecting specific fish species, the movement patterns and home range of these species of interest, must be first determined in order to establish the appropriate size of an MPA and its location [37, 38]. As some species of fish show ontogenetic differences in habitat use and movements [5, 39], an understanding of their behaviours across all life stages is essential.

Many recent studies have used acoustic telemetry to understand the movement dynamics of fish within MPAs. For example, luderick (*Girella tricuspidata*: Family Girellidae) were found to display strong site fidelity on shallow sub-tidal reefs within an MPA in New South Wales, Australia [40]. Similarly, dusky grouper (*Epinephelus marginatus*: Family Serranidae) were shown to display strong site fidelity and were regularly detected residing in a MPA for up to five years in the Azores [41, 42]. Small scale MPAs were found appropriate to protect the habitats and small home range of the comber (*Serranus cabrilla*: Family Serranidae) [43], and also the white sea-bream (*Diplodus sargus*: Family Sparidae) was observed to increase in abundance and biomass following the recent establishment of a small-sized MPA [44]. Eastern blue groper (*Achoerodus viridis*: Family Labridae) were shown to have smaller home ranges in no-take MPAs than in fished areas [45], whilst western blue groper (*Achoerodus gouldii*: Family Labridae) also displayed strong site fidelity and small home ranges within an MPA [46]. If protected areas are to be implemented to assist in the management of a particular species, then ideally the size of the protected area should be of sufficient size to cover core and home ranges, and that the species is known to display site fidelity to the proposed protected area.

The focus of this study is snapper (*Chrysophrys auratus*: (Family: Sparidae), a species of high value to commercial and recreational fisheries in Australia [47]. Its distribution in southern Australia encompasses the region from central Queensland to central Western Australia, including Tasmania [48], and it is also found in New Zealand [49]. It is considered a generalist predator [50] feeding on crustaceans, invertebrates and small fishes [51]. Adult fish can be found across a variety of habitats, particularly rocky reefs [49], whilst juveniles are known to inhabit estuaries and embayments [52–54]. *Chrysophrys auratus* in no-take MPAs have been found to occur in increased abundances [55–58] and display strong site fidelity [6]. Acoustic tagging studies in New Zealand have found that *C*. *auratus* displays high site fidelity with small overlapping home ranges [59], have extreme residency within a no-take marine reserve when compared to fished areas [60], and exhibit flexible behaviour, whereby their habitat utilisation can vary seasonally and between individuals [61]. Conversely, whilst the above mentioned studies indicate that *C*. *auratus* displays strong site fidelity and small home ranges, other studies have found that snapper can travel large distances. For example, adult *C*. *auratus* were recorded up to 370 km from initial capture location on the west coast of Australia [62] and adults have similarly been recorded travelling hundreds of kilometres in New Zealand [63].

Within New South Wales (NSW), Australia, there are six multiple-use MPAs that have been established “to conserve and protect marine biodiversity” [64] that include 115 separate no-take marine sanctuaries [65]. One of the species regularly highlighted when assessing the effectiveness of fishing exclusion from NSW MPAs is *C*. *auratus* [56, 58, 66] and they are considered to be one of three ‘indicator’ fish species for assessing MPA effectiveness in NSW [67]. However, there has been no published research in NSW on the movements or home range of this species. In particular, the movements of *C*. *auratus* within MPAs in NSW have not been described and it’s unknown whether they display site fidelity within no-take areas or if they move outside these zones into areas open to fishing. It is therefore necessary to determine of the degree of *C*. *auratus* site fidelity within protected areas or whether they are a transient species to MPA’s, as their use as an indicator species in monitoring programs for NSW marine parks will be enhanced if they can be demonstrated to display some form of site fidelity within the MPA. The aims of this study were therefore to: 1) determine the home and core ranges of juvenile and adult *C*. *auratus* within a small-sized MPA; 2) assess their site fidelity and movements through time, and; 3) determine their diel activity patterns. Information from this study will assist managers in future planning of MPAs to help manage fishery resources, as the size of MPAs needs to take into consideration the home range and site fidelity of the species of interest.

## Methods

### Study site

**Fig 1:**
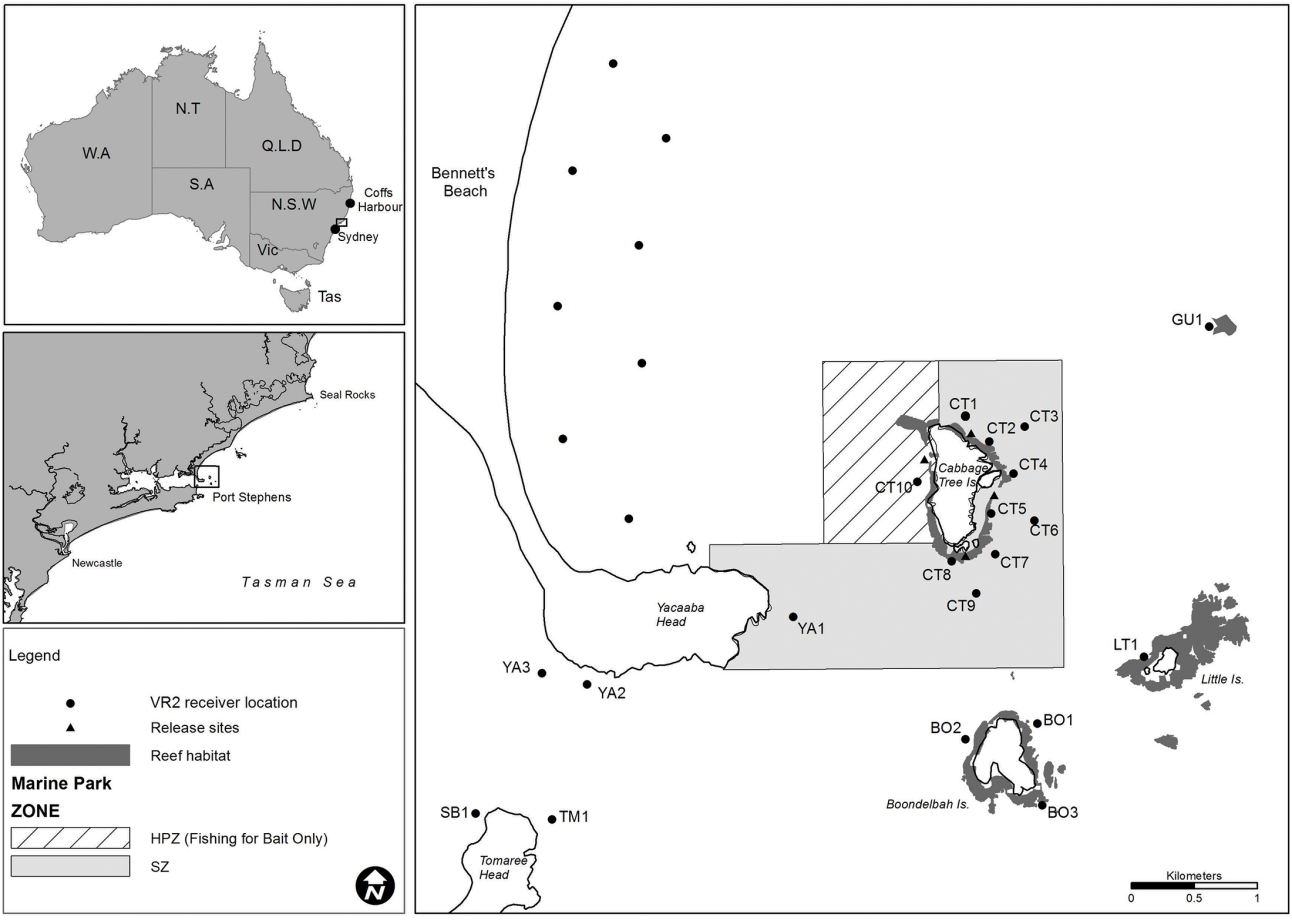
Location of Cabbage Tree Island in Port Stephens Great Lakes Marine Park and acoustic station array.

This study was conducted in the multiple-use Port Stephens-Great Lakes Marine Park (PSGLMP), approximately 980 km^2^ in size, located on the mid-north coast of NSW, Australia (Fig 1). The marine park was declared in December 2005 under the *NSW Marine Parks Act 1998* with implementation of the marine zoning plan commencing in April 2007. Within the marine park, there are various zones that provide different levels of protection. Sanctuary zones provide the highest level of protection with no extractive activities (i.e. fishing) permitted in these areas and represent 17.5% (171.6 km^2^) of the parks waters. The location for this study was Cabbage Tree Island (32°41'14.95"S 152°13'46.85"E) which is situated 1.5 km off the coast and is in close proximity to the mouth of the Port Stephens estuary (Fig 1). The waters surrounding Cabbage Tree Island are predominantly sanctuary zone (3.69 km^2^), whilst the western side of the island is classified a restricted habitat protection purpose zone (HPZ: 1.31 km^2^). Within the HPZ, fishing is permitted for only three bait fish species (yellowtail scad *Trachurus novaezelandiae*: Family Carangidae, slimy mackerel *Scomber australasicus*: Family Scombridae and eastern sea garfish *Hyporhamphus australis*: Family Hemiramphidae) (Fig 1). The reef surrounding Cabbage Tree Island is dominated by urchin-grazed barrens habitat which extends down to depths of 20 m, with the reef edge finishing in close proximity to the island (< 50 m) and the shallower sections (< 10 m) dominated by kelp (*Ecklonia radiata*: Family: Lessoniaceae) habitat.

### Receiver array and tagging procedures

An array of 18 acoustic receivers (VR2W, Vemco http://www.vemco.com) was established for this study. Ten receivers were deployed around Cabbage Tree Island with the other receivers covering nearby reefs and islands, whilst the entrance to the Port Stephens estuary was also ‘gated’ to detect if any *C*. *auratus* that migrated into the estuarine environment. Additionally, eight receivers were deployed along Bennetts Beach (Hawks Nest) as part of a juvenile white shark (*Carcharodon carcharias*) monitoring program [68, 69] (Fig 1) and these were also checked for any *C*. *auratus* detections. The AATAMS database that maintains acoustic receiver data for the Australian region, and lists the location of the AATAMS receiver network (http://imos.org.au/aatams.html), was also searched for *C*. *auratus* detections from the date tagged until September 2014. Deployments within the sanctuary zone were positioned to ensure that any tagged *C*. *auratus* within the Cabbage Tree Island sanctuary zone would be detected when present. Range testing was undertaken to assess the detection range of the receivers [70] using continuous ping acoustic tags on a mooring buoy and then listening at 100 m intervals out to 500 m. It was found that the minimum detection range for the receivers in the poorest sea conditions (run out low-tide with poor visibility and large swell) was approximately 400 m. Acoustic receivers were swapped over approximately every 6 months with the data downloaded using VEMCO software and viewed in Microsoft excel.

Thirty *C*. *auratus* were captured during three days (2–4 November 2010) with rod and line using either J-style hooks baited with frozen Australian sardines (*Sardinops sagax*: Family Clupeidae) or southern calamari (*Sepioteuthis australis*: Family Loliginidae), or soft plastic lures (Berkley Gulp, Berkley USA). Fish ranged in size from 24.5–57.5 cm fork length (FL) and were captured from water depths of 5–20 m. Fight duration ranged from < 1 min up to ~4 min. Once landed, using a soft mesh landing net, the fish were held in an aerated 100 L holding tank with a constant flow-through seawater supply provided by a battery-operated bilge pump. Prior to surgery, the fish were transferred into an anaesthetic bath (50 mg.L−1 AQUI-S, AQUIS-S NZ, Wellington, New Zealand) until the opercular rate decreased, the fish failed to respond to stimuli and a loss of equilibrium occurred (~10 min).

Fish were placed in an operating cradle for surgery. Anaesthesia was maintained using a recirculating pump system from a sump tank (containing anaesthetic further diluted to 25 mg.L−1 concentration) located below the operating cradle which pumped water over the gills via a soft plastic tube inserted into the fishes mouth. The water exited via the opercula and drained back into the sump. Surgical skin preparation was achieved with a light iodine scrub (Betadine, Mundipharma B.V., Hoevelaken, Netherlands). A few scales were removed allowing a small horizontal incision (~20 mm long) to be made with a sterile scalpel blade. The incision was made just to one side of, and perpendicular to, the ventral midline roughly half way between the cloaca and the base of the ventral fins. An iodine-soaked Vemco V13 or V16 acoustic transmitter (pulse interval 120-180s) was then inserted directly into the coelomic cavity. The type of transmitter used was dependent on the size of the fish with V13 transmitters used in 17 fish (ranging in size from 24.5 to 38.2 cm FL) and V16 transmitters used in 13 larger fish (37.5–57.5 cm FL). The antibiotic oxytetracycline hydrochloride (Sigma-Aldrich, Ballerup, Denmark) was also intracoelomically administered prior to incision closure at a dose of 75 mg.kg-1 body weight (length-weight relationship for *C*. *auratus* taken from (47). Skin and body wall closure was undertaken using 3/0 Monosyn^®^ glyconate absorbable monofilament suture material (B. Braun Australia P/L, Bella Vista, NSW, Australia) using two single interrupted sutures. Fish were also marked with a single external dart tag (Hallprint, Hallprint Pty Ltd, Hindmarsh Valley, South Australia; 85 mm long, 2 mm dia.) inserted into the dorsal musculature and secured between the dorsal pterygiophores. The external tags were used to identify fish carrying a transmitter if re-captured. Fish were then returned to the holding tank for recovery. During recovery, the fish were monitored for a return of reflexes and movement of the opercula, fins and body in a coordinated fashion (~10–40 min). Once the fish were swimming in an upright position with the same tail strength as when they were first caught, they were released at the surface at their respective capture locations.

### Data analysis

The data were filtered to remove any potential ‘false detections’, that occur due to signal collisions or background noise [71], by removing any single detections from a fish that occurred on a specific receiver within a 24 h period [18]. Additionally, data from the first 36 h post-tagging was excluded due to potentially atypical behaviors as a result of the tagging [45, 53, 60].

#### Site fidelity

Site fidelity was quantified using a Residency Index (RI) [45, 72]. This was calculated as the number of days when a fish was detected on any of the receivers within the array (the 18 receivers established for this study) divided by the total number of days the full array was deployed (i.e. until 31 October 2011). A value of 0 indicates no residency and a value of 1 indicates permanent residency [45, 46]. Receiver CT02 was missing from 15 April to 13 May 2010. To determine if this affected the RI, the RI for each fish was calculated with this period included and excluded and compared using a paired Wilcoxon test. Since the RI was non-normally distributed, a Spearman’s correlation was used to test for an association between the length of the fish and the RI from the full array deployment. A Mann-Whitney was used to determine if the tag type (V13 vs V16) had an effect on the RI.

Fine-scale site fidelity (i.e. the level of movement around the receiver array), was quantified as: (i) the number of receivers a fish was detected on each day, and; (ii) using the Minimum Linear Dispersal (MLD) method [40, 73]. The MLD was defined as the distance between where the fish were released post-tagging and the furthest receiver that fish was detected on [40]. The distance between the tagging locations and each receiver were calculated in ArcGIS 9.3 (ESRI, California). For any points where land obstructed the direct route between them, tracks were generated to simulate the shortest route a fish could swim. A Spearman’s correlation was used to test if the MLD was associated with body size (fork length).

#### Diel activity

To determine the diurnal behaviour of *C*. *auratus* the proportion of detections recorded during the day and at night was calculated for each fish and compared using a paired Wilcoxon test. Day and night phases were determined using sunrise and sunset times from Australian Government Geoscience Australia (http://www.ga.gov.au/geodesy/astro/sunrise.jsp; accessed 3 March 2014). Diel activity patterns were quantified as the mean detection frequency for each hour for each fish. Multiple linear regression was used to test if there was a relationship between the mean detection frequency and the time of the day, body size or tag type.

#### Core and home range estimates

Estimated positions of the fish were determined during short-term centres of activity (COAs) following methods described in Simpfendorfer *et al*. [74]. An appropriate time interval (Δt), that allows a sufficient number of detections to be recorded on the more distant receivers but does not allow the fish to move too much, must be chosen to produce accurate results [74]. The ‘best’ Δt was selected by testing six different time intervals (10, 20, 30, 40, 50 and 60 mins) using methods described in Villegas-Rios *et al*. [72]. For this study, the ‘best’ Δt was 30 mins (number of receivers fish detected on per time interval: 2.1 ± 0.04; total number of detections per fish from all receivers per time interval: 11.0 ± 0.93).

The fish were categorized as ‘resident’ or ‘non-resident’ following [60], whereby resident fish that were detected > 65% of available half hour time bins. Only the resident fish provided sufficient information to perform a meaningful core and home-range analysis [60]. Kernel utilization distributions (KUDs) are commonly used to estimate the core and home range of an animal using the 50% and 95% contours, respectively [40, 60]. The half hourly COAs were used to calculate the core and home ranges of each *C*. *auratus* using the *adehabitatHR* package [75] in R [76]. Missing receivers would likely bias core/home ranges; therefore, only data when the full array was deployed were used in the estimations (4 November 2010 to 15 April 2011 and 13 May to 31 October 2011). Two core and home ranges were estimated: (i) for the entire study period (as specified above), and (ii) for the spawning and non-spawning seasons. *Chrysophrys auratus* are serial spawners [77], spawning batches of eggs daily [78] from August to October in New South Wales [79]. Therefore, August to October was defined as the spawning season and January to May as the non-spawning season (excluding November, December, June and July), to ensure no overlap of seasons.

Linear mixed effects models were used to determine if there was a significant difference in core/home ranges between: (i) maturity stage (juvenile/adult fish); (ii) the spawning and non-spawning seasons, and; (iii) spawning/non-spawning season according to maturity stage (i.e. an interaction between the terms). Tag type could not be included in the model as it was directly correlated with the maturity stage of the fish (Pearson’s correlation: r = 1.0). The unique fish identity code was used as a random effect. Fish under 35 cm (fork length) were classified as juveniles, which was the length at maturity for 95% of snapper caught off the NSW coast [79]. Data exploration was done following the protocol of Zuur et al. [80]. The linear mixed models were run using the R ‘lme4’ package [81]. The most parsimonious model structure was selected using the step-down protocol outlined in Zuur *et al*. [82]. Temporal correlation and variance structures [83] were used during the model selection process and their inclusion or exclusion in the model was based on Akaike’s Information Criterion for small sample sizes (AICc). Support for each model was measured using the differences in AICc (ΔAICc) where the ‘best’ model ΔAICc equals zero and ΔAICc of < 4 indicate models with considerable support [84]. If ΔAICc indicated support for more than one model, model averaging across normalized Akaike weights was conducted using the ‘MuMIn’ package in R [85]. Model validity was checked by visual examination of residual plots, plots of the standardized residuals versus theoretical quartiles (Q-Q plots) and checking the variance of the residuals for each level of the predicator variables [82]. The significance of each fixed effect predictor variable was estimated using a likelihood ratio test, whereby the model including a particular term was compared with a model excluding that term and its interactions, using the R ‘anova()’ function. Tukey’s pairwise comparisons were used if a multi-level covariate was included in the ‘best’ model (i.e. spawning/non-spawning season and maturity stage interaction term) and were done using the ‘multcomp’ package in R [86].

#### Daily day/night space use estimates

Linear mixed effects models were used to determine if there was a relationship between *C*. *auratus* daily day/night space use and various environmental (time of day (day/night), water temperature, moon illumination, and spawning/non-spawning season) and biological (maturity stage) variables. Again, tag type could not be included as it was directly correlated with maturity stage. Space use (95% KUDs) was calculated for day/night-time of each day within the spawning and non-spawning seasons for each of the fish considered resident. The day or night phases were determined using methods previously described (see Methods: *Diel activity*). Moon illumination data was obtained from the United States Naval Observatory Astronomical Applications Department (http://aa.usno.navy.mil/data/docs/MoonPhase). A Vemco minilog was deployed within the study area from March 2011 to record water temperature (SEACAMS data). A log-10 transformation was applied to the daily day/night space use estimates to improve assumptions of normality and homogeneity. Model selection and the importance of each term were carried out using the same methods as described above (Methods: *Core and home range estimates*).

### Ethics statement

This research was undertaken in accordance with the ethical standards of the NSW DPI Animal Care and Ethics Committee (Permit No. 09/07).

## Results

Fourteen of the 30 tagged fish were considered adults (> 35 cm, fork length) and sixteen were considered juveniles (Table 1). Two fish were never detected after being tagged (fish #4 and #16) and two others were only detected for 7 and 8 days (fish #5 and #9), respectively, post release. It is unknown whether these fish left the area, were predated on or died as a result of the tagging procedure or that the tags malfunctioned; however, they were not detected on any other receivers away from Cabbage Tree Island. Given the uncertainty about the fate of these fish, they were removed from subsequent analyses. The residency index (RI) for the remaining fish varied between 0.1 and 1.0 with a mean RI of 0.83 (i.e. fish were detected 83% of days from 36 hour post tagging to the 31 October 2011; SE = 0.05) (Table 1).

**Table 1:** Summary of 30 *Chrysophrys auratus* tagged within the Port Stephen’s-Great Lakes marine park.

Fish ID	FL (cm)	Date tagged	Last detected	Area tagged	Tagged in SZ or HPZ	RI	Resident
1	32.9	3/11/10	17/11/11	North	SZ	0.99	Y
2	34.8	2/11/10	22/04/11	East	SZ	0.41	N
3	33.6	2/11/10	17/11/11	East	SZ	1.00	Y
4	25.5	2/11/10		East	SZ	0.00	N
5	25.9	3/11/10	10/11/10	South	SZ	0.02	N
6	26.7	3/11/10	09/11/11	South	SZ	0.62	N
7	25.6	3/11/10	13/11/11	South	SZ	0.98	Y
8	24.5	3/11/10	17/11/11	South	SZ	0.99	Y
9	25.4	2/11/10	10/11/10	East	SZ	0.01	N
10	26.3	3/11/10	16/06/11	South	SZ	0.58	N
11	29.6	3/11/10	18/11/11	South	SZ	0.99	Y
12	25.9	3/11/10	18/11/11	South	SZ	0.99	Y
13	27.4	2/11/10	28/06/11	East	SZ	0.62	N
14	27.7	3/11/10	16/11/11	South	SZ	0.99	Y
15	34.1	3/11/10	18/11/11	South	SZ	0.99	Y
16	26.4	3/11/10		South	SZ	0.00	N
17	38.2	4/11/10	18/11/11	West	HPZ	0.92	N
18	37.8	3/11/10	01/01/12	North	HPZ	0.89	N
19	53.0	2/11/10	27/02/14	East	SZ	1.00	Y
20	57.5	3/11/10	02/12/13	North	HPZ	0.99	Y
21	42.1	3/11/10	10/02/11	West	HPZ	0.29	N
22	40.7	4/11/10	16/01/14	East	SZ	0.99	Y
23	38.6	4/11/10	03/03/12	South	SZ	0.99	Y
24	39.5	3/11/10	03/01/14	North	HPZ	0.99	Y
25	37.5	4/11/10	04/03/14	East	SZ	0.99	Y
26	49.6	3/11/10	08/12/10	West	HPZ	0.10	N
27	55.3	4/11/10	05/04/14	West	HPZ	0.99	Y
28	48.3	4/11/10	06/04/14	West	HPZ	0.99	N
29	48.6	4/11/10	11/02/12	West	HPZ	0.83	N
30	46.0	4/11/10	01/06/11	West	HPZ	0.54	N

FL = fork length. SZ = sanctuary zone. HPZ = habitat protection zone. Resident fish were those detected > 65% of available half hour time bins.

Nine fish continued to be detected regularly after this time period when the majority of the receivers were removed and six fish continued to be detected at the site in 2014 (Table 1). No fish were detected remaining at the site in acoustic surveys undertaken in October 2014. The longest duration that a fish was continuously recorded at Cabbage Tree Island, and not recorded at any other sites, was 1249 days (fish #28). There was a significant difference in the RI calculated that included and excluded dates when a receiver was missing from the array (paired Wilcoxon test: p-value = 0.04). Therefore, the RI, and the subsequent analyses, only used data when the full array was available, i.e. until October 2011. Body size did not significantly affect the RI (Spearman correlation: rho = 0.25; p = 0.20) nor did the tag type (Mann-Whitney: W = 84.5, p = 0.38).

The number of receivers a *C*. *auratus* was detected on per day ranged between 0 and 10 (4.2 ± 0.42; mean ± SE). The maximum distance between the tagging location to the furthest receiver a fish was detected on (MLD) ranged between 785–2311 m (1302 ± 82 m; mean ± SE). One fish (#29) was only ever detected on the receiver closest to its release location (90 m). Three fish were detected on receivers away from Cabbage Tree Island. One fish (#6) was detected on the Little Island receiver (~ 1.7 km from Cabbage Tree Island; Fig 1) from June to July 2011, and then again one day in November 2011 before it was not detected again. Another fish (#20) was detected at Little Island on a single day in July 2011 but this fish was also detected on the receivers around Cabbage Tree Island before and after being detected at Little Island. Data from the AATAMS network indicated that a single larger adult (#30: 46 cm when tagged) travelled a very large distance. It was last detected at Cabbage Tree Island on 01/06/2011, then recorded at Seal Rocks (32°27'49.93"S 152°33'9.77"E) on 21–23 June 2011 and last recorded at Coffs Harbour (30°17'17.27"S 153°11'15.04"E) on 16–17 July 2011. Over a period of 46 days, this fish moved a total distance of ~ 290 kms. There was no association between MLD and fish size (Spearman’s correlation: rho = 0.16, p = 0.39).

**Fig 2:**
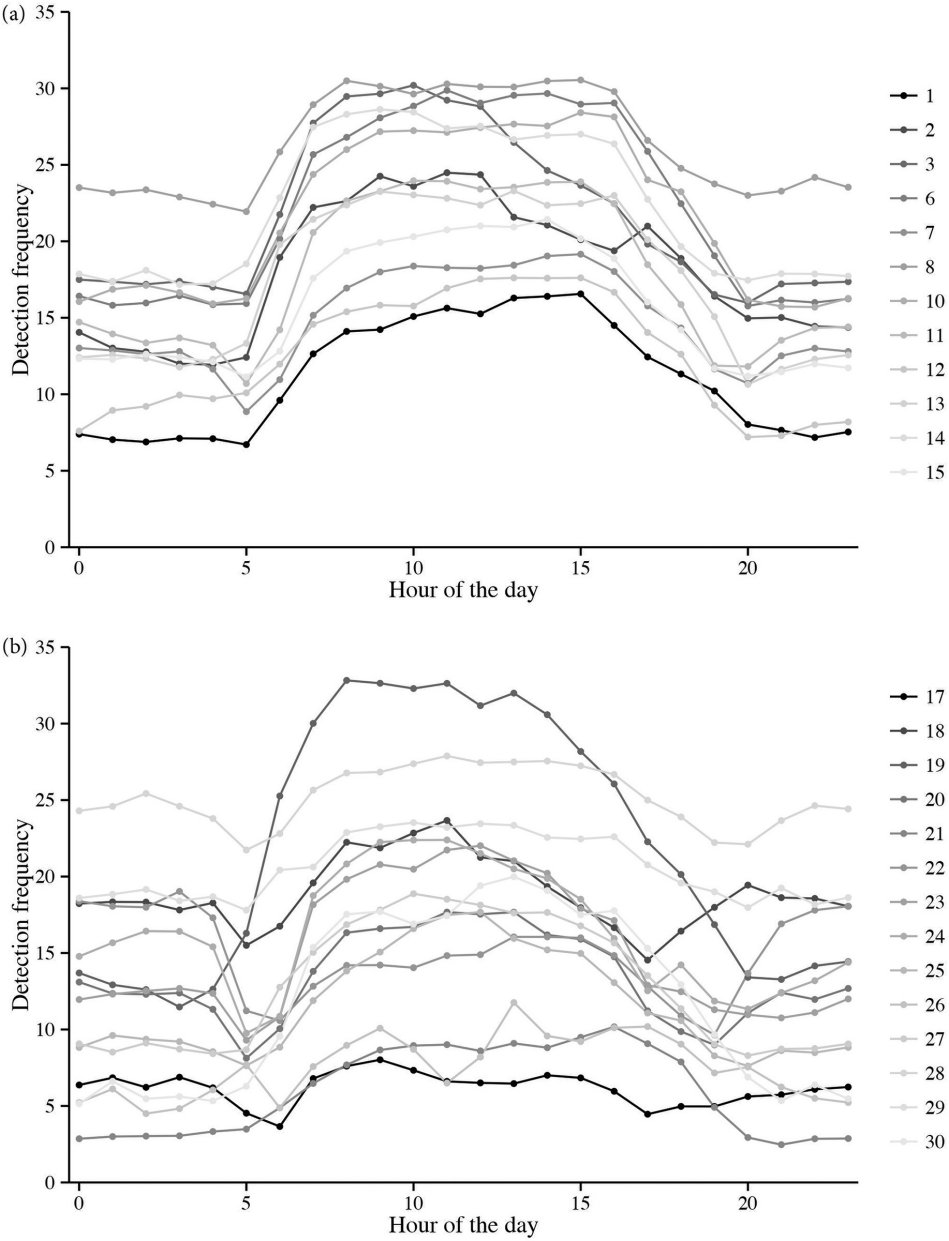
Diel activity patterns of tagged *Chrysophrys auratus*. a-b indicate the maturity stage of the tagged fish: a = juvenile; b = adult. Hour of the day is represented by a 24 hour clock.

*Chrysophrys auratus* displayed strong diurnal behaviour and were detected significantly more during the day than at night (paired Wilcoxon test: p-value < 0.001), with an average of 67% of the detections occurring during the daylight hours (range: day = 53–85%; night: 14–47%) (Fig 2a and 2b). There was no significant difference in the detection frequency between the different hours of the day or tag type (multiple linear regression: p-values = > 0.05); however, there was a significant difference depending on the size of the fish (multiple linear regression: p-value < 0.001) with the mean detection frequency decreasing for larger fish (Multiple linear regression: estimate = -0.18). Although, this model only accounted for a very small amount of deviance observed (R^2^ = 0.05).

**Fig 3:**
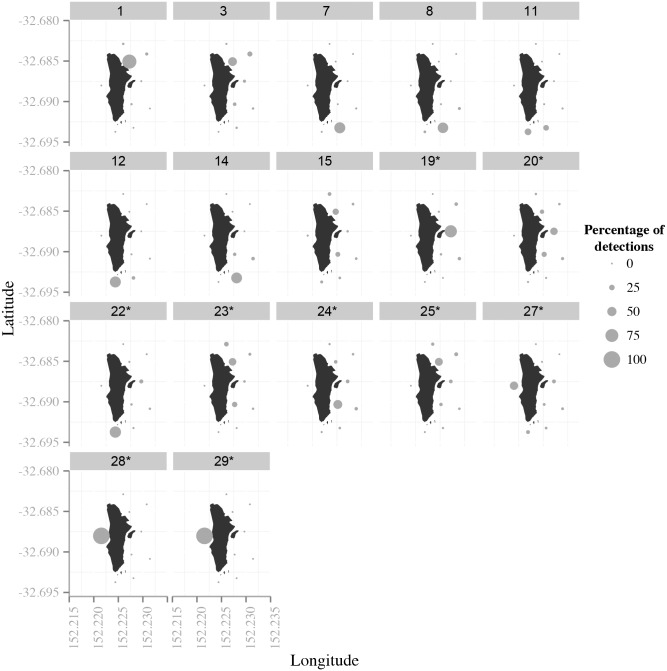
Bubble plots showing the percentage of detections at each receiver for those fish considered to be resident fish around Cabbage Tree Island (fish that were detected > 65% of available half hour time bins). * indicates adult fish.

**Fig 4:**
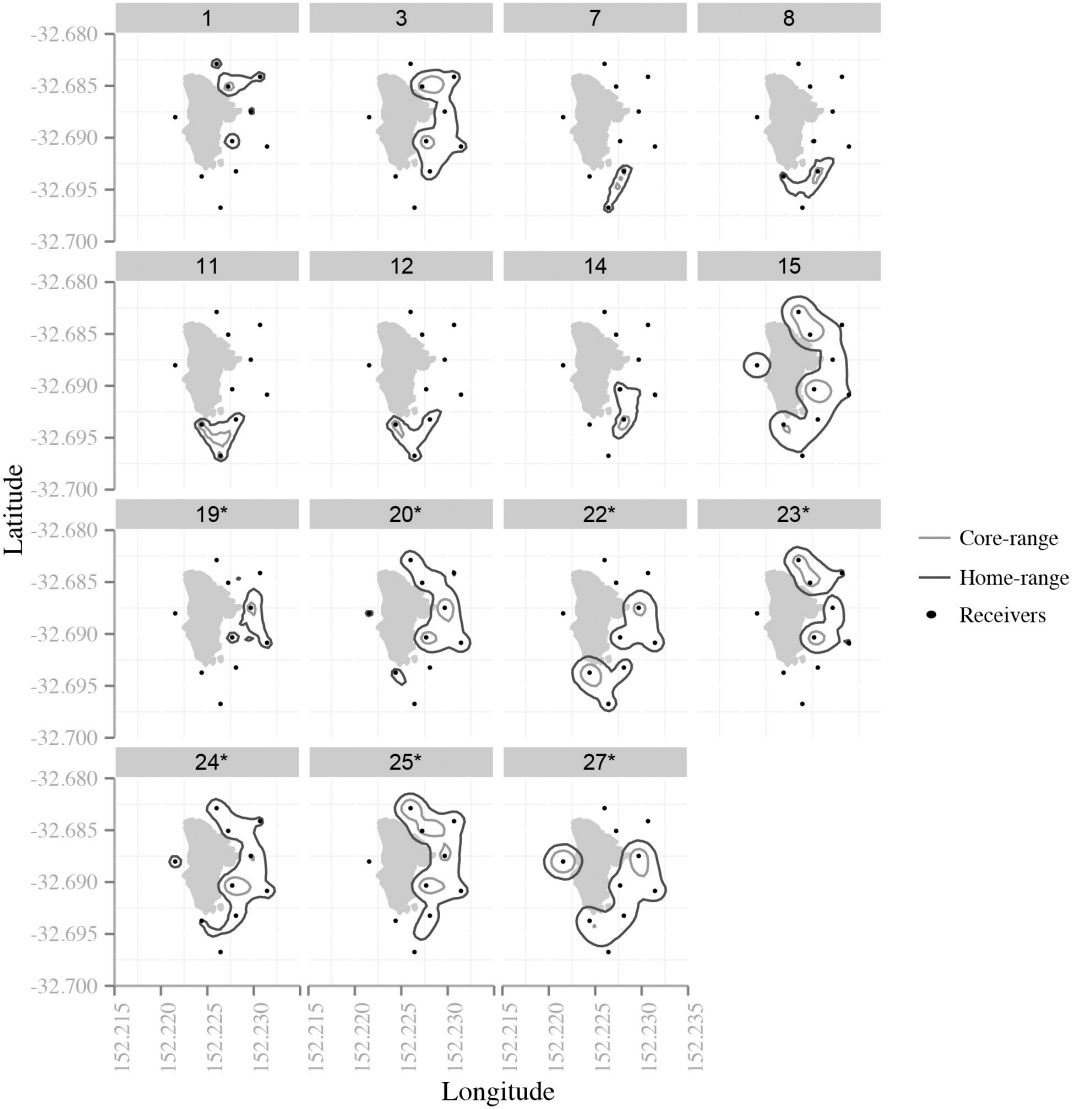
Core and home range estimates for resident *Chrysophrys auratus*. * indicates adult fish. Fish 28 and 29 are not included as they were only detected on one receiver.

Four snapper, in addition to the four fish suspected to have died, were excluded from COAs estimations as they were only detected on a single receiver within each half hourly period (including fish #28 and #29). A mean of 11832 ± 779 COAs were estimated for the remaining 22 fish (*n* = 6 juveniles); however, only 15 fish were categorised as ‘residents’ (Table 1; Fig 3). Overall core and home ranges of these fish varied between 0.01–0.12 km^2^ and 0.05–0.70 km^2^, respectively (median core range: 0.04; median home range: 0.33) (Fig 4).

**Fig 5:**
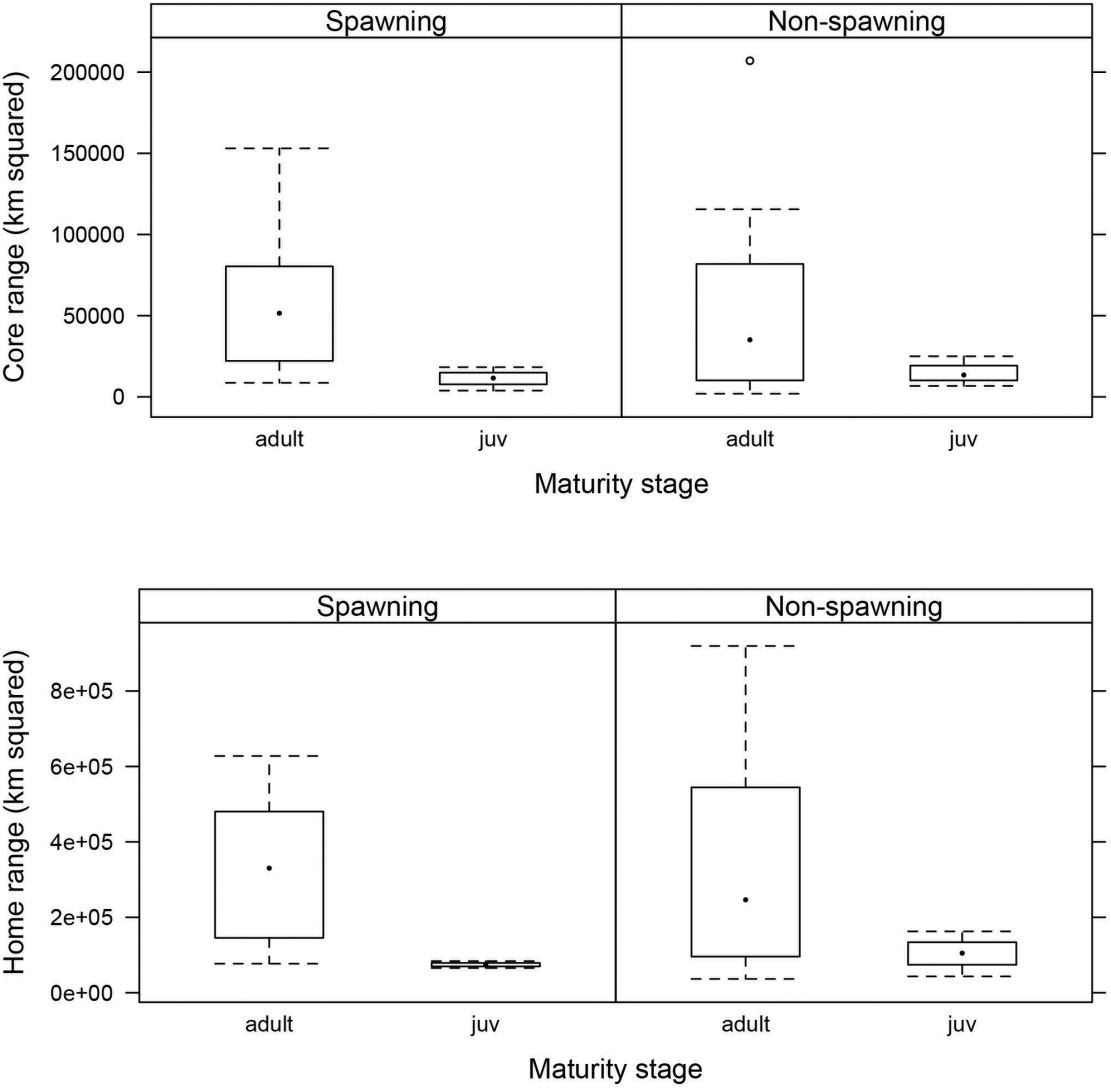
Core and home range estimates of resident *Chrysophrys auratus* for the spawning and non-spawning season, grouped by maturity stage. The dot inside the box indicates the median; the box indicates the first and third quartile and the whiskers indicate the minimum and maximum values.

Model selection on core ranges produced four model candidates with a ΔAICc < 4 (Table 2). The maturity stage was the best model predictor and had a relative importance of 0.41. The juvenile fish had smaller core-ranges than the adults (Fig 5); however, this difference was not significant (intercept estimate: 10.4, juvenile estimate: -0.68, likelihood ratio: p-value = 0.15). There was also no significant difference between maturity stages spawning/non-spawning season (likelihood ratio: p-value = 0.41; relative importance: 0.26). The interaction between maturity stage and spawning/non-spawning season was not included in any of the ‘best’ models (Table 2), therefore, there was no difference between the seasons according to maturity stage.

**Table 2:** The ‘best’ models (ΔAIC_c_ < 4) for linear mixed effects models on core and home ranges.

Model	df	AICc	ΔAICc	Model weight
**Core ranges (50% KUD)**				
~ NULL	3	96.47	0.00	0.43
~ maturity stage	4	97.10	0.63	0.31
~ spawn	4	98.45	1.99	0.16
~ maturity stage + spawn	5	99.31	2.84	0.10
**Home range (95% KUD)**				
~ maturity stage	4	75.37	0.00	0.49
~ NULL	3	76.52	1.15	0.28
~ maturity stage + spawn	5	77.84	2.47	0.14
~ spawn	4	78.76	3.39	0.09

Fixed effects: *spawn* = spawning/non-spawning season; *maturity stage* = adult/juvenile. The unique fish identity code was used as the random effect.

There were also four model candidates with a ΔAICc < 4 produced from model selection on home range estimates (Table 2). Like the core ranges, the maturity stage was the best model predictor (relative importance: 0.63), and although the juveniles had smaller home-ranges (Fig 5), this difference was not significant (likelihood ratio: p-value = 0.5). Spawning/non-spawning season was also not significant (likelihood ratio: p-value = 0.51; relative importance: 0.23). Again, the interaction between maturity stage and spawning/non-spawning season was not included in any of the ‘best’ models (Table 2).

Model selection on daily day/night space use estimates produced two candidate models: the full model (i.e. all the variables; model weight = 0.72) and the full model without water temperature (model weight = 0.28). The best model predictors were: *maturity stage* according to spawning/non-spawning season (i.e. the interaction between *maturity stage* and spawning/non-spawning season), day/night-time, day length and moon illumination and all had a relative importance of 1.0. Juveniles had significantly smaller daily day/night space use than the adults in the non-spawning season (p-value = 0.04) but not in the spawning season (p-value = 0.41). Adult fish had significantly smaller daily day/night space use in the spawning season (p-value < 0.001) while the juveniles’ space use was significantly smaller in the non-spawning season (p-value < 0.001). The daily day/night space use was also significantly smaller at night and increased with increasing moon illumination (both p-values < 0.001). There was no significant difference for water temperature (p-value = 0.79; relative importance: 0.28).

## Discussion

This acoustic tagging study represents the first detailed assessment of movement patterns of *C*. *auratus* within a MPA on the east coast of Australia. It demonstrated that a high proportion of *C*. *auratus* displayed strong site fidelity within a no-take sanctuary zone around an offshore island, and that one fish undertook a large migration northwards along the NSW coast. Whilst this MPA was not specifically designed to protect *C*. *auratus*, it is clear that even at a small size, that the MPA can provide benefits to both juvenile and adult fish which display strong site connection, through the removal of incidental and targeted fishing pressures.

### Home and core range

This study found that most *C*. *auratus* maintained small home ranges within the study area and that there was no significant difference in core or home ranges between juveniles and adults. Whilst there was no significant difference in core or home ranges between adults and juveniles, the adults were found to have much larger core and home ranges but it was much more variable (Fig 5). It was also shown that individual *C*. *auratus* display bimodal space use, similar to the findings of [59]; however, the reasons for these bimodal patterns are unknown. The habitats around Cabbage Tree Island have not been mapped so it’s not possible to make assumptions in relation to habitat availability and reef topography (depth and gradient). These bimodal differences may be influenced by other factors such as higher concentrations of prey or increased shelter, or are related to foraging or social interaction. These findings of fish having small home ranges within a no-fishing area are similar to the results of a study in New Zealand that found that *C*. *auratus* tagged in an area open to fishing had home ranges with more than one main area of use and spanned a greater area overall, when compared with fish tagged in a non-fishing area, which only had one main area of use and hence a much smaller home range [60]. However, it is not known if the small home ranges within the no fishing reserve are a direct result of the protection status, or could possibly be driven by other factors such as reef topography or habitat characteristics, such as the presence of kelp as alluded to in Parsons *et al*. [60].

It has been previously suggested that protected areas (no-fishing) can lead to extreme residency in fish through modification of individual behaviours or through the removal of selective exploitation [60]. Modification of fish behaviour within sanctuary zones has also been hypothesised to occur in response to the increased presence of scuba divers and associated feeding [45]; however, the sanctuary zone around Cabbage Tree Island is not considered a popular scuba diving location and is infrequently visited by local dive operators (Harasti *per obs*).

### Site fidelity / Movements

It was found that *C*. *auratus* displayed very strong site fidelity to the rocky reefs surrounding Cabbage Tree Island. Twenty-six of the thirty tagged fish (87%) remained continuously at the site for the first 6 months of the study and were not detected on any adjacent reefs, islands or on the AATMAS acoustic receiver network along the NSW coast. This includes the four fish that were considered to have either died following tagging or the tags malfunctioned, but it is feasible that these fish may have migrated to areas where no acoustic receivers were present. No fish were detected moving into the adjacent Port Stephens estuary or along the nearby beaches. The size of the fish and the original capture location did not influence the residency index (RI) and only three fish were detected on stations outside the Cabbage Tree Island array. The RI for *C*. *auratus* at the site was considered high (0.83) when compared to other fish species [87, 88], and *C*. *auratus* have been shown to display strong site fidelity to a range of habitats including rocky reefs [6, 59, 60], embayments [89, 90] and estuarine systems [53]. In contrast to these findings, *C*. *auratus* have also been found to display significant movements from their tagging site [62, 89] and site fidelity is considered to vary between individuals and seasons [61]. Similar to the findings of Egli & Babcock [61], some *C*. *auratus* were detected moving outside the Cabbage Tree Island sanctuary zone to adjacent reefs and islands. Such movements demonstrate some limited potential for *C*. *auratus* to spill over into fishable areas, yet with the majority of fish remaining protected from harvesting within the MPA. Additionally, site fidelity of *C*. *auratus* may be affected by the size of the actual protected area, but could also be influenced by other factors within the protected area such as reef topography and available habitat types [60, 91]. *Chrysophys auratus* are known to occur in a range of marine habitats, ranging from rocky reefs [63], soft corals [52] to macro algal habitats, such as kelp [60, 91]. The types of habitats, and their associated abundance, available in a protected area need to be taken into consideration for any planning of protected areas for the purposes of conserving *C*. *auratus*. Whilst this study provides an indication of the home range sizes of *C*. *auratus*, it would be inadequate to declare a protected area for *C*. *auratus* unless there was sufficient suitable habitat available for the species.

A single tagged adult *C*. *auratus* in the present study was shown to have travelled a large distance within a short time frame (~290 kms over 46 days), an average rate of ~ 6.3 km per day. *Chrysophrys auratus* have been previously shown to be capable of travelling large distances along the east coast of Australia. A NSW Fisheries tagging program in the 1980’s recorded an individual *C*. *auratus* travelling up to 420 km over a period of 173 days (from Sydney to Arrawarra), whilst another fish moved a considerable distance from Coffs Harbour to Mooloolaba in Queensland (430 km) in 597 days (NSW DPI *unpublished data*). In Queensland, a single *C*. *auratus* was observed moving 290 km over a period of 6 months [89]; however, this fish was not acoustically tagged so it’s unknown if it arrived at its recapture location earlier than 6 months. It is possible that the individual from the present study that travelled ~ 290 km in only 46 days did so as part of a pre-spawning migration; however, the role pre-spawning migrations may have in the dynamics of *C*. *auratus* spawning along eastern Australia requires considerable further work. It is also feasible that the fish that undertook this northern migration was a transient fish to the island, rather than a resident fish, as it has been shown that different stocks of *C*. *auratus* can display either transient or resident behaviour [62].

### Diurnal activity and day/night space use

It was clear from diel activity patterns that *C*. *auratus* were more active during the day than at night; however, there was no particular hour of day when they were most active. Similarly, it has been shown in an estuarine study on *C*. *auratus* in New Zealand that their activity was predominantly diurnal [53]. One factor that could be confounding the strong diurnal activity is increased ambient noise at night, which may have a negative effect on detection ability [92]. However, the potential increase of marine noise at night interfering with acoustic detections was not assessed in this study. The reef surrounding Cabbage Tree Island is dominated by large complex rocky boulder habitat which contains many potential refugia in which a fish could hide. It is therefore likely that during the night, *C*. *auratus* take shelter within the reef which in turn reduces detections through the acoustic array. Indeed, observations made scuba diving at night at this site has revealed *C*. *auratus* hiding in amongst the complex rocky boulder habitat (Harasti *per obs*).

### MPA implications

Whilst MPAs in NSW are not fishery management tools and have not been specifically designed to protect *C*. *auratus* from fishing, it is clear that individuals of this species would benefit from no take sanctuary zones as a result of their small home range and strong site fidelity. It has been shown for another sparidae species with known small home ranges and high site fidelity (the white sea-bream *Diplodus sargus*), that both abundance and biomass increased following the establishment of a small MPA [44]. The cause of these increased residency patterns with MPAs could be driven by increased prey abundance following the removal of fishing, habitat characteristics, local population densities, and con-specific densities, or the structure of mating systems [60, 61, 93, 94]. Given that the abundance of *C*. *auratus* has been to shown to increase in protected areas within the PSGLMP over a short period of time [67], this increase in con-specific density could potentially be the main driver in their decreased home ranges within the protected area; however, additional acoustic telemetry research at other protected and non-protected sites is warranted to investigate this further.

When the strong site fidelity of *C*. *auratus* is combined with observations that the abundance of *C*. *auratus* is higher within no-take areas than outside [6, 57, 67], the use of *C*. *auratus* as an ‘indicator’ species in long term monitoring programs for temperate marine parks is validated. This could be used to help assess the effectiveness of marine park zoning compliance in NSW, as the overall size and relative abundance of *C*. *auratus* should increase over time in areas protected from fishing.
